# Chronic exposure to yttrium induced cell apoptosis in the testis by mediating Ca^2+^/IP3R1/CaMKII signaling

**DOI:** 10.3389/fpubh.2023.1104195

**Published:** 2023-01-30

**Authors:** Zhehao Liu, Yechun Ding, Shuchun Xie, Yaqiong Hu, Hai Xiao, Xia Liu, Xiaona Fan

**Affiliations:** ^1^School of Public Health and Health Management, Gannan Medical University, Ganzhou, China; ^2^College of Pharmacy, Gannan Medical University, Ganzhou, China; ^3^School of Basic Medicine, Gannan Medical University, Ganzhou, China; ^4^Department of Pathology, First Affiliated Hospital of Gannan Medical University, Ganzhou, China; ^5^Key Laboratory of Prevention and Treatment Cardiovascular and Cerebrovascular Disease of Ministry of Education of Gannan Medical University, Ganzhou, China

**Keywords:** rare earth element, testis, YCl_3_, IP3R1, CaMKII

## Abstract

**Introduction:**

Environmental pollutants, such as rare earth elements, affect human health and particularly induce reproductive system injury. Yttrium (Y), one of the most widely used heavy rare earth elements, has been reported the cytotoxicity. However, the biological effects of Y^3+^ in the human body are largely unknown.

**Methods:**

To further investigate the effects of Y on the reproductive system, *in vivo* (rat models) and *in vitro* studies were performed. Histopathological and immunohistochemical examination were conducted, and western blotting assays were performed to detect the protein expression. TUNEL/DAPI staining were used to detect cell apoptosis, and the intracellular calcium concentrations were also determined.

**Results:**

Long-term exposure to YCl_3_ in rats produced significant pathological changes. YCl_3_ treatment could induce cell apoptosis *in vivo* and *in vitro*. In addition, YCl_3_ enhanced the concentration of cytosolic Ca^2+^ and up regulated the expression of IP3R1/CaMKII axis in Leydig cells. However, inhibition of IP3R1 and CaMKII with 2-APB and KN93, respectively, could reverse these effects.

**Conclusion:**

Long-term exposure to yttrium could induce testicular injury by stimulating cell apoptosis, which might be associated with activation of Ca^2+^/IP3R1/CaMKII axis in Leydig cells.

## Introduction

Rare earth elements have been comprehensively employed in agricultural, industrial, and medicinal fields. Yttrium (Y) is one of the most widely used heavy rare earth elements. Most studies on Y in the medical field are focusing on its beneficial characteristics for the improvement of laser system, which is employed for insemination *in vitro* (1). Chemically, Y may irritate to eyes, skin, and respiratory system. Inhalation of dust containing Y can lead to occupational pneumoconiosis, and YCl_3_ may detrimentally stimulate the mucous membrane of the eyes. The safety and efficacy of Y90 in patients with lung shunt fraction >15% have been discussed. Some patients may have non-specific pulmonary symptoms, such as cough, shortness of breath, wheezing in the 1-year post-Y90 (2). Y has been demonstrated to potentially produce liver damage with inflammation, necrosis, and portal fibrosis (3). The ion Y^3+^ can be accessible in the south of Ganzhou city in China. Our previous study indicated that Y^3+^ can induce neuronal cell death by triggering apoptotic pathway in rats (4). However, the biological effects of Y^3+^ in the human body are largely unknown.

Yttrium oxide nanoparticles have been recently to shown to alleviate the reproductive toxicity induced by silver nanoparticle (5). However, it has been reported that the rare earth elements can be accumulated in the human body and produce detrimental effects in a dose-dependent manner (6). The rare earth elements can be accumulated in the liver, bone, and lungs (7). It has been demonstrated that the rare earth elements show reproductive toxicity through multiple interventions, including agricultural and industrial pathways (8). The interaction between the Tb (IV)-NR complex and sperm DNA gene information has been verified (9). Cerium oxide nanoparticles can be accumulated in the testis, inducing sperm DNA damage and reproductive toxicity (10). The biological effects of Y^3+^ on the reproductive system still need to be clearly elucidated.

Ca^2+^, a second messenger, has been involved in various cell functions. The critical roles of Ca^2+^ in the testis have been demonstrated (11). The imbalanced homeostasis of Ca^2+^ may contribute to cell death. Particularly, Ca^2+^ can sensitize the signal in the pro-apoptotic transition of the mitochondria and stimulate cell apoptosis. Ca^2+^ signaling includes reactive proteins, such as calmodulin (CaM) and Ca^2+^/CaM-dependent protein kinase II (CaMKII) (12). Inositol 1,4,5-triphosphate receptor 1 (IP3R1) maintains intracellular Ca^2+^ homeostasis (13). Increased activity of the Ca^2+^-mediated IP3R1/CaMKII pathway has been associated with cell apoptosis (14). Many rare earth elements, such as Eu^3+^ (15) and La^3+^ (16), may have similar biological activities by interacting with Ca^2+^-related channels. Whether Y^3+^ exhibits reproductive toxicity and induces cell apoptosis by activating the IP3R1/CaMKII pathway is still unclear. In this article, we will mainly investigate the biological roles of Y^3+^ in the testis.

## Materials and methods

### General

This project (GMU2017012) was approved by the Ethics Committee of Gannan Medical University, according to the Declaration of Helsinki Principles. The study was conducted in accordance with the internationally accepted principles for laboratory animal use and care in the European Community guidelines (EEC Directive of 1986; 86/609/EEC). Thirty-six-week-old male rats were kept in an adaptive circumstance in an SPF-grade room with a 12 h light/dark cycle (Temperature: 21 −23°C; humidity: 45%−55%) for 1 week before further experiments. All rats were free to access water and food. Yttrium chloride (YCl_3_, purity ≥ 99.99%) was purchased from Sigma-Aldrich (St. Louis, USA).

### Animal experiments

Rats were randomly divided into three groups of 10 rats each, including the negative control (NC) group (free access to blank water) and YCl_3_-treated groups (free access to water with 12 and 24 mmol/L of YCl_3_, respectively) (4). After 6 months, all rats were sacrificed. The testicular tissues were obtained for weight measurement, histopathological and immunohistochemical examination, and protein extraction.

### Gonadosomatic index

The testicular weight and the body weight of each rat were determined. The gonadosomatic index was reached by the formula: gonadosomatic index = the testicular weight /the body weight × 100% (17).

### Determination of semen quality

The cauda epididymis was harvested and minced in the physiological saline at 36°C to prepare a sperm suspension. The sperms (100 sperms) moving in a straight line were counted using a blood cell counting plate, which was preheated at 36°C. To detect the concentration of sperm, the suspension (100 μl) was killed by a preheated water bath at 60°C. The sperm was counted using a blood cell counting plate. To examine the sperm deformity rate, the suspension (500 μl) was smeared and fixed with methanol. The sperm deformity was observed using a microscope.

### Determination of serum testosterone

A testosterone ELISA kit (Cat. no. KB3117, BOYAO, Shanghai, China) was employed to detect the concentrations of serum testosterone in rats. The procedures were conducted according to the instructions recommended by the kit manufacturers.

### Histopathological and immunohistochemical examination

The collected testicular tissues were fixed with 4% paraformaldehyde. Then, they were embedded in the paraffin. The 5-μm thickness of sections was sliced and then stained with hematoxylin-eosin (H&E). Light microscopy was used to observe the pathological changes. For the immunohistochemical examination, the testicular tissues embedded in paraffin were sectioned for 5-μm thickness, deparaffinized in xylene, and rehydrated with different graded concentrations of ethanol on glass slides. The tissues were then interacted with by hydrogen peroxide for 10 min and incubated the primary antibodies (1:100) for 6 min and horseradish peroxidase (HRP)-conjugated secondary antibody for 8 min, respectively. After further incubation with 3,3-diaminobenzidine (DAB) for 10 min. Light microscopy was used to observe the immunized proteins (18, 19).

### Cell viability assays

The CCK8 kit (Cat.no.C0038) (Beyotime, Shanghai, China) was used for testing the viability of Leydig cells (purchased from Procell Life Science&Technology Co. Ltd, Wuhan, China), according to the manufacturer's instructions. Leydig cells were grown in 96-well plates at a density of 5,000 cells/well for 24 h. Different concentrations of YCl_3_ (0, 3, 10, 30, 100, and 300 μM) were used to pretreat the Leydig cells for 24 h. Then, CCK-8 solution (10 μl) was added into each well and incubated at 37°C for 4 h. After that, the absorbance was detected at the wavelength of 450 nm by using a microplate system (Leica microsystems, Germany).

### TUNEL/DAPI staining

TUNEL and DAPI staining kits were purchased from Abcam (Cambridge, MA, USA). Leydig cells were cultured and treated with YCl_3_ for 48 h. Cells were harvested and then fixed with 4% paraformaldehyde. The apoptosis was analysis according to the instructions recommended by the manufacturer. The fluorescent images were observed using a Leica microsystem, and the florescence intensity was determined using ImageJ (an open-source image processing package).

### Western blot

The total protein was harvested, and the protein concentrations were detected by BCA protein assay kit (Beyotime). Each sample (30 μg) was subjected to SDS-PAGE and then transferred onto PVDF membranes. After being blocked, the membranes were co-incubated with the primary antibodies at 4°C overnight against IP3R1 (1: 1,000 dilution; Sigma), CaMKII (1: 1,000 dilution; Sigma), p-CaMKII (1: 1,000 dilution; Sigma), Bcl-2 (1: 1,000 dilution; Sigma), caspase-3 (1: 1,000 dilution; Sigma), cleaved-caspase-3 (1: 1,000 dilution; Sigma), and β-actin (1: 1,000 dilution; Sigma). Then, the secondary antibody conjugated with peroxidase (1: 5,000 dilution; Sigma) was employed. Protein bands were detected using the enhanced chemiluminescence detection system.

### Quantification of cytosolic calcium concentrations

The experimental procedures were conducted according to the instructions of the kit's manufacturer (HL10153.1, Shanghai Haling Biological Technology, Shanghai, China). Leydig cells were incubated with Rhod-2/AM for 1 h at 37°C. Analysis was conducted by a fluorescence microplate reader (Varioskanlux, Thermo Fisher Scientific, Waltham, MA, USA) with the parameters, including the wavelength of 550 nm (excitation) and 590 nm (emission).

### Statistical analysis

All experiments were performed three times independently and data are expressed as the mean ± standard deviation (SD). SPSS 20.0 software was employed for statistical analysis. One-way ANOVA and Turkey's *post hoc* test were used to analyze the differences between multiple groups. *P* < 0.05 indicated a significant difference statistically.

## Results

### Chronic exposure to YCl_3_ produced damage to the rat testis

**Figure 1:**
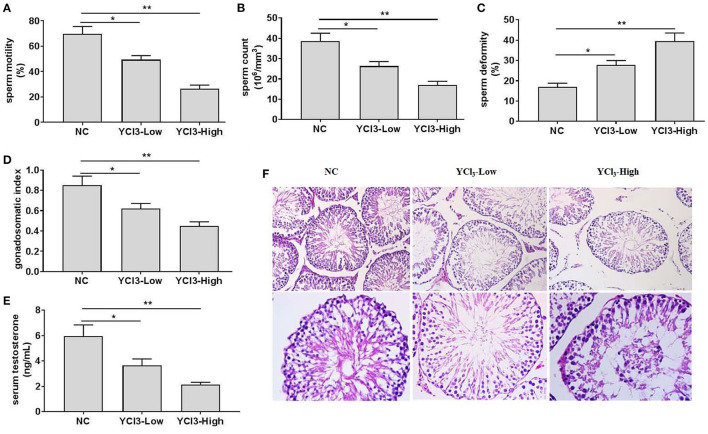
The effects of chronic exposure to YCl_3_ on the rat testis (*n* = 10) were investigated. The sperm quality, including sperm motility **(A)**, sperm count **(B)**, and sperm deformity **(C)** was detected. The gonadosomatic index **(D)** and the serum testosterone level **(E)** were measured. The histopathological examination **(F)** of testicular tissues [magnification ×200 (up) and ×400 (down)] by HE staining was conducted. *P < 0.05, **P < 0.01. NC, negative control; YCl_3_-Low, 12 mmol/L YCl_3_; YCl_3_-High, 24 mmol/L YCl_3_.

Rats were free to access water containing 12 and 24 mmol/L of YCl_3_, respectively, for 6 months, and the chronic effects of YCl_3_ on the testis in rats were investigated. The results showed that YCl_3_ could dose-dependently reduce the semen quality in rats, as indicated by decreased sperm motility (Figure 1A) and concentrations (Figure 1B) and increased sperm deformity (Figure 1C). In addition, the gonadosomatic index (Figure 1D) and the levels of serum testosterone (Figure 1E) were also decreased by YCl_3_ treatment in a dose-dependent manner. Histopathological examination by HE staining (Figure 1F) in the YCl_3_-treated group indicated that the arrangement of spermatogenic cells at all levels was loose and disordered and the number of mature spermatozoa was reduced, compared with those in the negative control group. Furthermore, the anatomic structure of seminiferous tubules was damaged and the distance among the seminiferous tubules was enlarged. These suggested that YCl_3_ treatment induced significant toxicity and damage to the testis.

### YCl_3_-induced testis damage was associated with increased apoptosis

**Figure 2:**
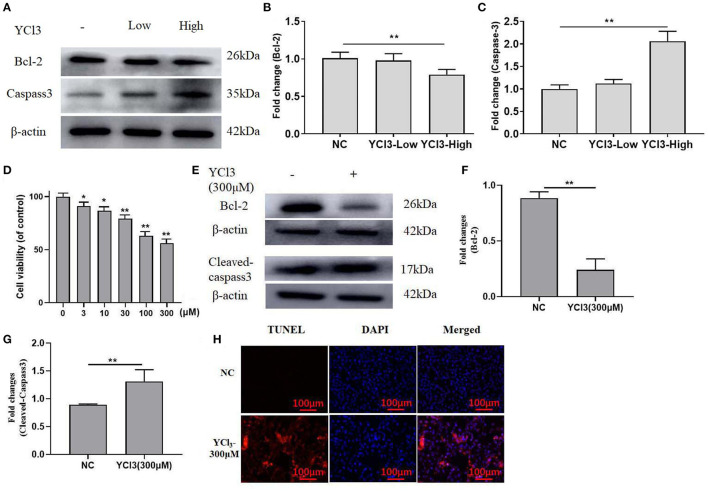
The effects of YCl_3_ on cell apoptosis. The protein expression of Bcl-2 **(A, B)** and caspase-3 **(A, C)** in the testis was detected by Western blot. **(D)** CCK8 assays were detected for the effects of YCl_3_ on the cell viability, the statistical difference was compared to the NC group (0 μM). Western blot assays were conducted for determination of Bcl-2 **(E, F)**, and cleaved-caspase-3 **(E, G)** protein expression. TUNEL/DAPI assays were conducted for apoptosis detection **(H)**. *P < 0.05, **P < 0.01. NC, negative control; YCl_3_-Low, 12 mmol/L YCl_3_; YCl_3_-High, 24 mmol/L YCl_3_.

To further investigate how YCl_3_ treatment induced testis damage, the protein expression of Bcl-2 and caspase-3 in the testis organs was detected. YCl_3_ treatment could down regulate Bcl-2 expression (Figures 2A, B) and up regulate caspase-3 expression (Figures 2A, C), indicating induction of apoptosis. In addition, the *in vitro* study on the cytotoxicity of YCl_3_ on the cultured Leydig cells was detected. The results from the cellular viability detection showed that YCl_3_ at the doses of 300 μM indicated almost the greatest cytotoxicity (Figure 2D). Additionally, the expression of cleaved caspase-3 was up regulated, and the expression of Bcl-2 was down regulated (Figures 2E–G). In the TUNEL/DAPI staining assays, YCl_3_ significantly increased cell apoptosis (Figure 2H). Collectively, the induction of testis damage by YCl_3_ was associated with increased apoptosis.

### YCl_3_ induced cell apoptosis by up regulating the expression of IP3R1

**Figure 3:**
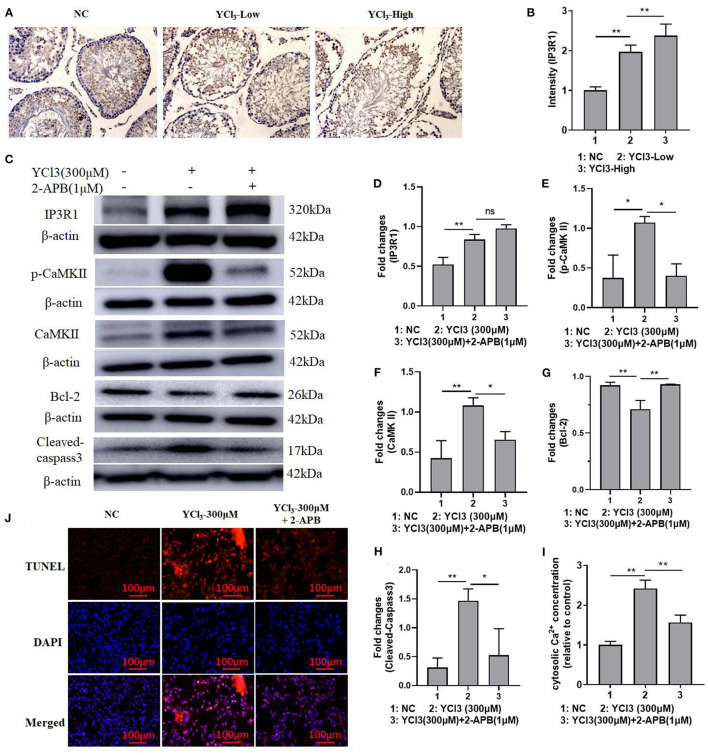
The roles of IP3R1 in the induction of cell apoptosis by YCl_3_. The immunohistochemical study (magnification ×200) of IP3R1 in YCl_3_-treated rat testicular tissue was tested **(A)**, and it is quantified by intensity measurement **(B)**. Western blot assays were conducted for determination of IP3R1 **(C, D)**, p-CaMKII **(C, E)**, CaMKII **(C, F)**, Bcl-2 **(C, G)**, and cleaved-caspase-3 **(C, H)** protein expression. The cytosolic Ca^2+^ concentration was detected by the kits **(I)**. TUNEL/DAPI assays were conducted for apoptosis detection **(J)**. *P < 0.05, **P < 0.01. NC, negative control; YCl_3_-Low, 12 mmol/L YCl_3_; YCl_3_-High, 24 mmol/L YCl_3_.

To further explore the possible mechanism of YCl_3_ in the regulation of apoptosis, the expression of IP3R1 was detected. The *in vivo* immunohistochemical study (Figures 3A, B) showed that the expression of IP3R1 in YCl_3_-treated rats was increased. In YCl_3_-treated Leydig cells *in vitro*, the protein expression of IP3R1, p-CaMKII, and CaMKII (Figures 3C–F) was up regulated. The protein expression of Bcl-2 (Figures 3C, G) was increased, and the expression of cleaved caspase-3 (Figures 3C, H) was decreased. In addition, the concentration of cytosolic Ca^2+^ (Figure 3I) was significantly enhanced. The apoptosis rate (Figure 3J) was elevated. The stimulating effects of YCl_3_ were blocked by co-treatment with IP3R1 inhibitor 2-APB (1 μM). These suggested that YCl_3_ might induce cell apoptosis by up regulating the expression of Ca^2+^/IP3R1 signaling in Leydig cells.

### YCl_3_ induced cell apoptosis by up regulating the activity of the Ca^2+^/CaMKII axis

**Figure 4:**
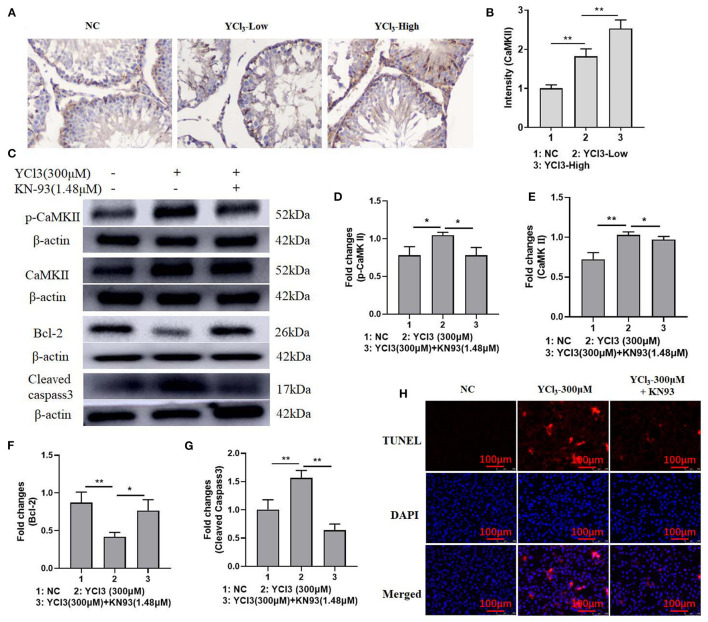
The effects of YCl_3_ on the activity of Ca^2+^/CaMKII axis. **(A)** The immunohistochemical study (magnification ×200) **(A)** of CaMKII in YCl_3_-treated rat testicular tissue was tested, and it is quantified by intensity measurement **(B)**. Western blot assays were conducted for determination of p-CaMKII **(C, D)**, CaMKII **(C, E)**, Bcl-2 **(C, F)**, and cleaved-caspase-3 **(C, G)** protein expression. TUNEL/DAPI assays were conducted for apoptosis detection **(H)**. *P < 0.05, **P < 0.01. NC, negative control; YCl_3_-Low, 12 mmol/L YCl_3_; YCl_3_-High, 24 mmol/L YCl_3_.

To further explore the possible mechanism of YCl_3_ in the regulation of cell apoptosis, the activity of CaMKII was determined. The expression of CaMKII (Figures 4A, B) in YCl_3_-treated rats was investigated by an *in vivo* immunohistochemical study, and it was significantly increased. The expression of CaMKII and p-CaMKII in YCl_3_-treated Leydig cells *in vitro* was also up regulated. After co-treatment with CaMKII inhibitor KN93 (1.48 μM), the increased expression of p-CaMKII and cleaved caspase-3 and the decreased expression of Bcl-2 were reversed (Figures 4C–G). Similarly, the changes in cell apoptosis (Figure 4H) were rescued. Collectively, YCl_3_ induced cell apoptosis by up regulating the activity of CaMKII in Leydig cells.

## Discussion

Many rare earth elements in the drinking water and foods have been reported to significantly affect our health (8, 20). Long-term exposure to the rare earth elements leading to accumulation in the body has caused negative outcomes (21). In this study, we found that long-term exposure to the rare earth element yttrium produced reproductive toxicity by damaging the testis. The pathological changes might be association with increased cell apoptosis induced by yttrium treatment, which could activate the Ca^2+^/IP3R1/CaMKII axis in Leydig cells.

Recently, male infertility increases dramatically. Exposure to environmental pollutants might be the attributing factor (22). It has been reported that cadmium (Cd) has become a serious environmental pollutant to induce testis injury by inducing cell apoptosis (23). Lanthanum, one of the important rare earth elements, has been reported to induce severe pathological changes in testicular tissues and apoptosis in spermatogenic cells by induction of oxidative stress and reduction of anti-oxidant enzyme expression, such as NRF2/HO-1 and PI3K/AKT signaling pathways (24). Gadolinium has been demonstrated to be accumulated in the testis organ and leads to its malfunctions, as shown by the increased loss of spermatozoa and accumulation of immature germinal cells in the seminiferous tubule lumen (25). Consistently, our study showed that long-term exposure to yttrium resulted in increased cell apoptosis and testicular malfunctions.

The underlying mechanism of the rare earth elements in inducing pathological changes may be complex. Cadmium has been assumed to pass through the cellular membrane by the transporters for Ca, Fe, Mn, and Zn, due to their similar chemical and physical properties (26). Many channels, such as voltage-dependent cation channels (VDCCs) and depolarization-activated calcium channels (DACCs), responsible for Ca^2+^ transportation have been reported to transport Cd^2+^. These channels are featured as non-selective cation channels (27). Cadmium induces neurological apoptosis by promoting mitochondrial distribution and mitochondrial fission, which might be associated with overload of calcium and excessive activation Ca^2+^ signaling pathway (28). Long-term exposure to the trivalent lanthanum and gadolinium rare earth elements promotes their internalization and activation of intracellular Ca^2+^/calmodulin signaling (29). Consistently, our study also found that treatment with YCl_3_ induced increased cytosolic Ca^2+^ concentration. In addition, the expression of IP3R1/CaMKII was also increased. Inhibition of IP3R1 and CaMKII, respectively, might reverse the detrimental effects of YCl_3_ on Leydig cells, indicating that Yttrium induced cell apoptosis by activating Ca^2+^/IP3R1/CaMKII signaling pathway.

However, there are limitations to this study. In the rat models, the administration doses are not scientific strict because rats were not ensured to receive their supposed amount of YCl_3_. Fortunately, the study *in vivo* was designed for 6 months, and this makes it possible to diminish the discrepancy. In addition, free access to water containing YCl_3_ can alleviate the discomfort induced by gavage or injection. More efforts should be made in exploring the mechanism of yttrium in passing through the blood testicular barrier. In addition, the effects of YCl_3_ after inhibition of calcium channels on Leydig cells should be investigated.

## Conclusion

Long-term exposure to the rare earth element yttrium induced testicular injury. Specifically, Y could reduce the semen quality, gonadosomatic index, and serum testosterone levels. In addition, Y might increase cell apoptosis *in vivo* and *in vitro* and enhance the concentration of cytosolic Ca^2+^. The underlying mechanism of Y in inducing testicular injury might be associated with activation of Ca^2+^/IP3R1/CaMKII signaling pathway. After inhibition of IP3R1 and CaMKII activities, the negative effects of Y on Leydig cells might be ameliorated. This might provide a potential therapeutic strategy for treating those patients with Y-induced testis damage.

## Data availability statement

The original contributions presented in the study are included in the article/supplementary material, further inquiries can be directed to the corresponding author.

## Ethics statement

The animal study was reviewed and approved by the Ethics Committee of Gannan Medical University.

## Author contributions

Conceptualization: XF. Investigation: ZL and YD. Writing—original draft preparation: SX and YH. Review and editing: HX and XL. All authors have read and agreed to the published version of the manuscript.
