# Supplementary material for: Chronic exposure to yttrium induced cell apoptosis in the testis by mediating Ca2+/IP3R1/CaMKII signaling
Source: Front Public Health. 2023 Jan 30;11:1104195. doi: 10.3389/fpubh.2023.1104195 (PMC9923002; doi:10.3389/fpubh.2023.1104195)
Supplement: Supplementary file 1 [file Data_Sheet_1.ZIP › original data/original gels.pdf]

Figure 1

Bcl-2

Repeat1

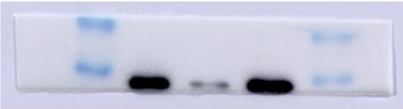

Bcl-2

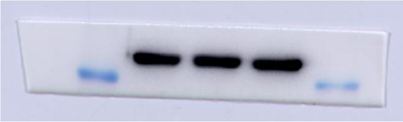

β-actin

Repeat2

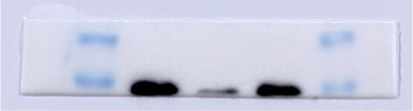

Bcl-2

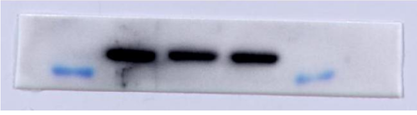

β-actin

Repeat3

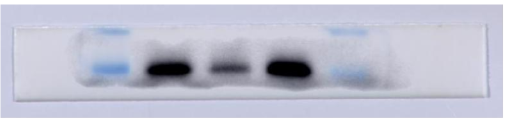

Bcl-2

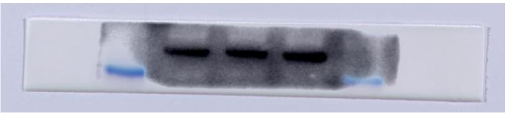

β-actin

Cleaved-Caspass3

Repeat1

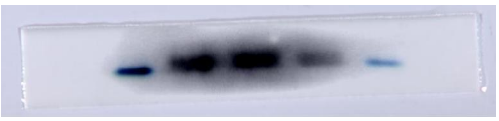

Cleaved-Caspass3

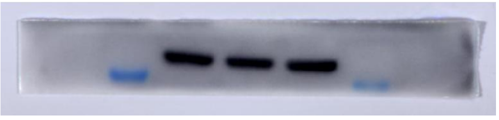

β-actin

Repeat2

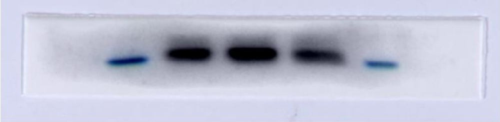

Cleaved-Caspass3

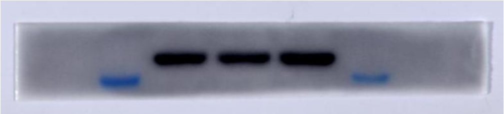

β-actin

Repeat3

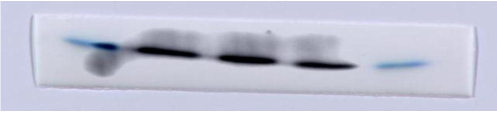

Cleaved-Caspass3

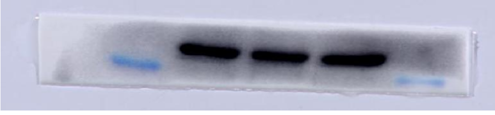

β-actin

Figure 2

Bcl-2

Repeat1

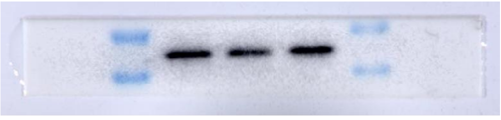

Bcl-2

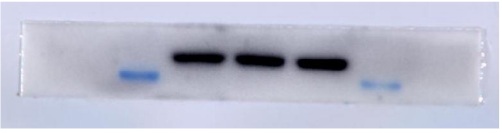

β-actin

Repeat2

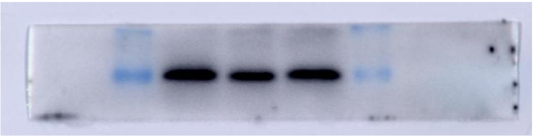

Bcl-2

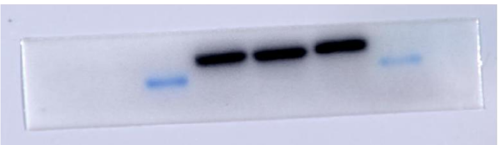

β-actin

Repeat3

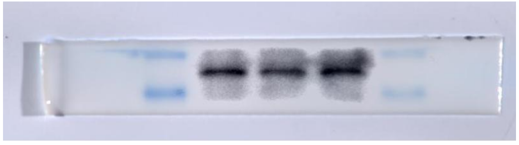

Bcl-2

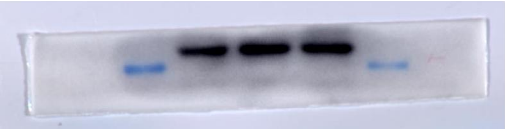

β-actin

Cleaved-Caspass3

Repeat1

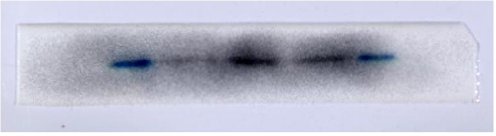

Cleaved-Caspass3

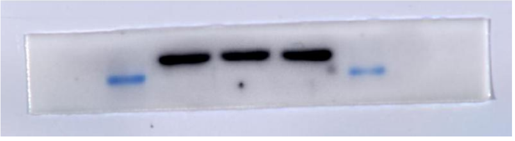

β-actin

Repeat2

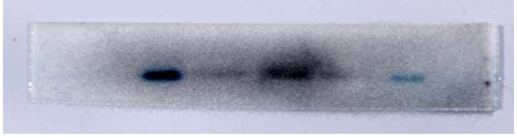

Cleaved-Caspass3

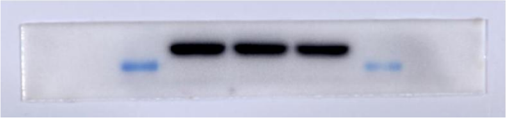

β-actin

Repeat3

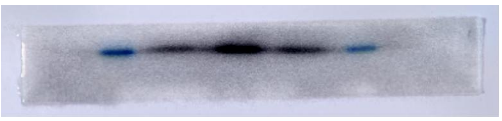

Cleaved-Caspass3

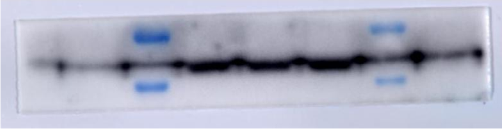

β-actin

IP3R1

Repeat1

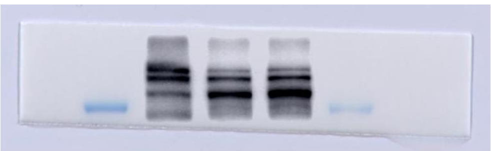

Ip3r

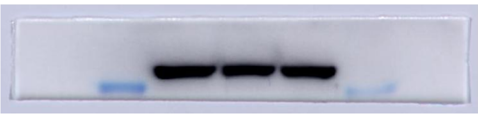

β-actin

Repeat2

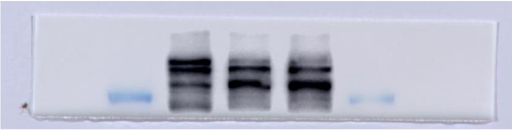

Ip3r

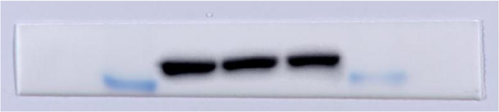

β-actin

Repeat3

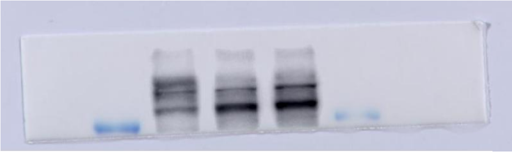

Ip3r

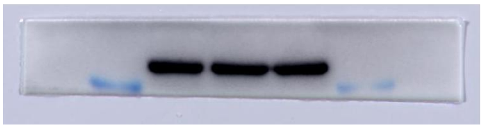

β-actin

p-CaMK II

Repeat1

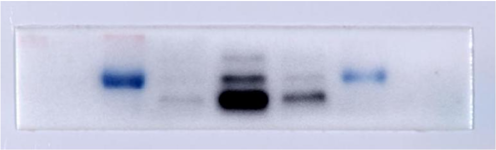

p-CaMK II

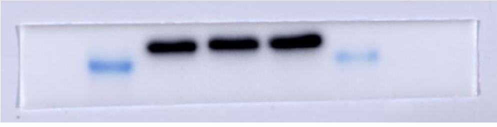

β-actin

Repeat2

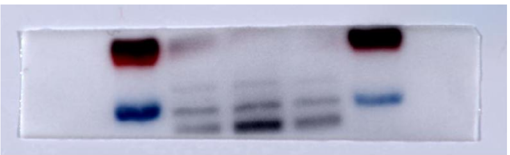

p-CaMK II

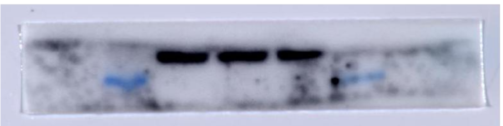

β-actin

Repeat3

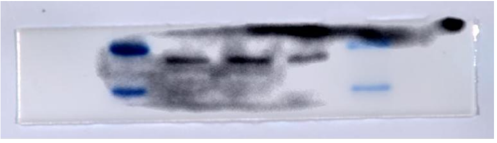

p-CaMK II

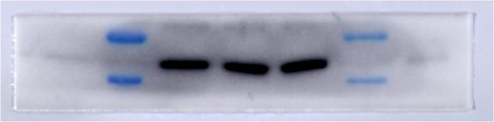

β-actin

CaMK II

Repeat1

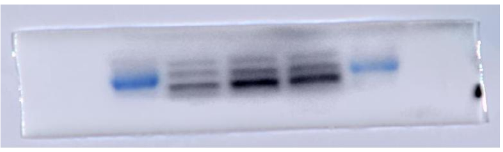

CaMK II

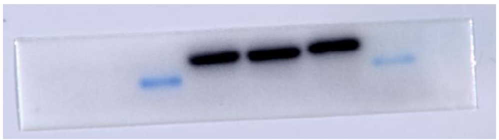

β-actin

Repeat2

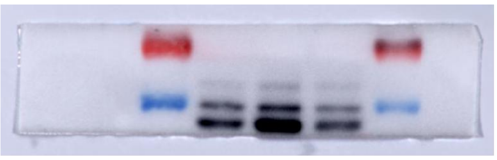

CaMK II

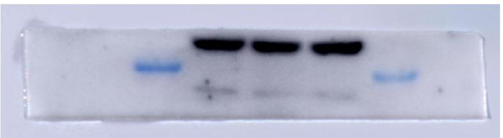

β-actin

Repeat3

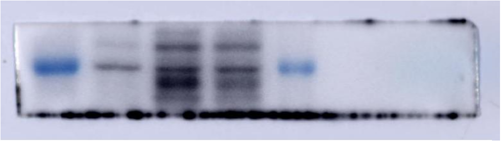

CaMK II

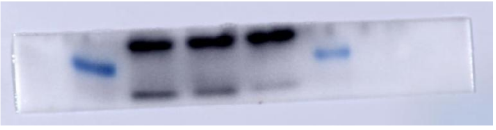

β-actin

Figure 3

Bcl-2

Repeat1

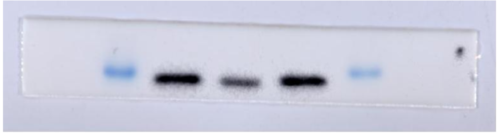

Bcl-2

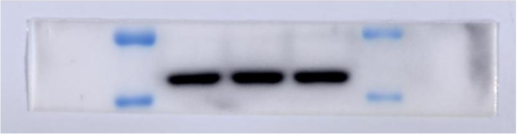

β-actin

Repeat2

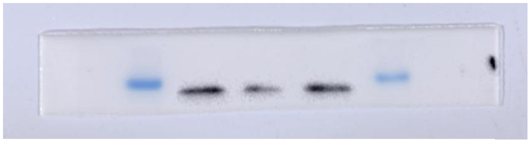

Bcl-2

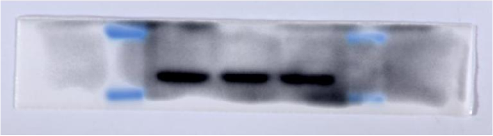

β-actin

Repeat3

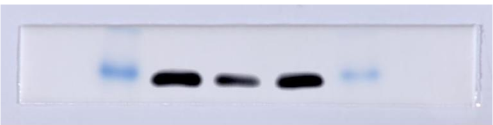

Bcl-2

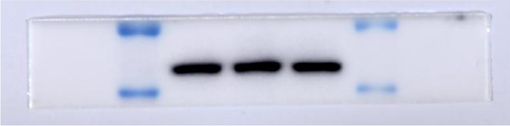

β-actin

Cleaved-Caspass3

Repeat1

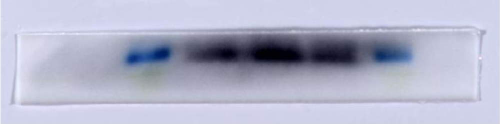

Cleaved-Caspass3

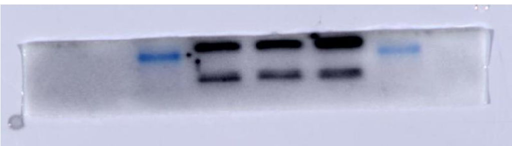

β-actin

Repeat2

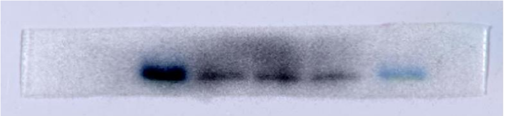

Cleaved-Caspass3

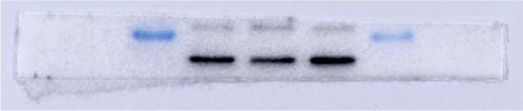

β-actin

Repeat3

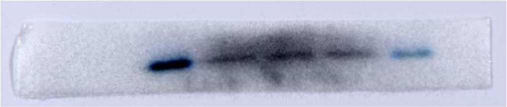

Cleaved-Caspass3

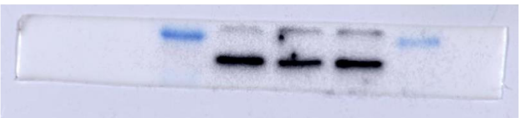

β-actin

p-CaMK II

Repeat1

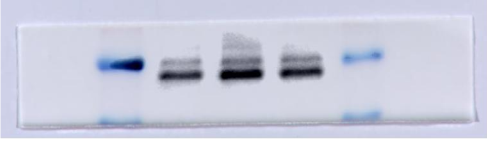

p-CaMK II

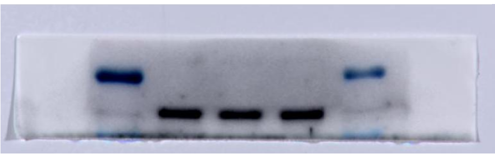

β-actin

Repeat2

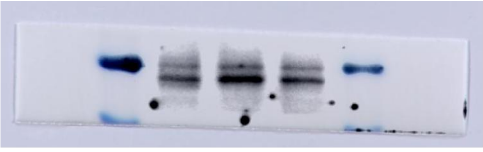

p-CaMK II

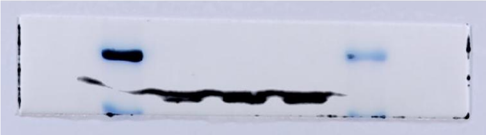

β-actin

Repeat3

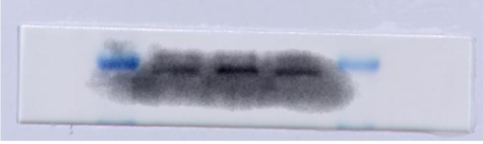

p-CaMK II

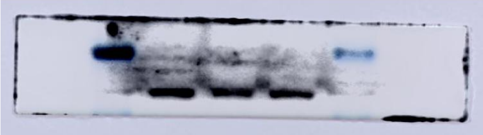

β-actin

CaMK II

Repeat1

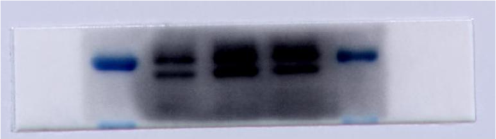

CaMK II

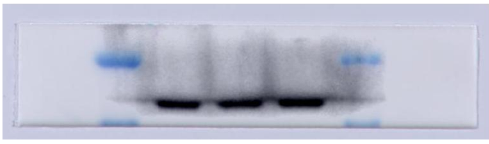

β-actin

Repeat2

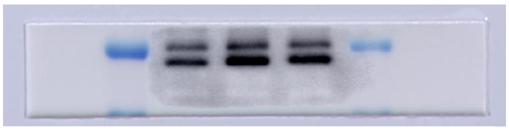

CaMK II

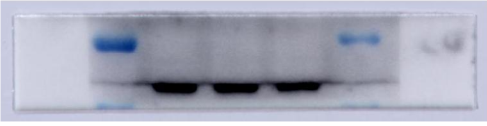

β-actin

Repeat3

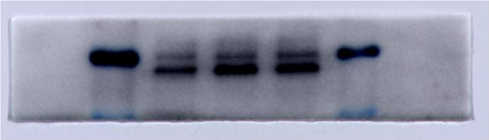

CaMK II

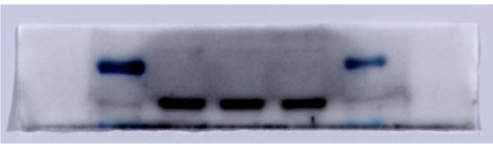

β-actin
