# Supplementary material for: Chronic exposure to yttrium induced cell apoptosis in the testis by mediating Ca2+/IP3R1/CaMKII signaling
Source: Front Public Health. 2023 Jan 30;11:1104195. doi: 10.3389/fpubh.2023.1104195 (PMC9923002; doi:10.3389/fpubh.2023.1104195)
Supplement: Supplementary file 1 [file Data_Sheet_1.ZIP › original data/ppt.pptx]

## Slide 1
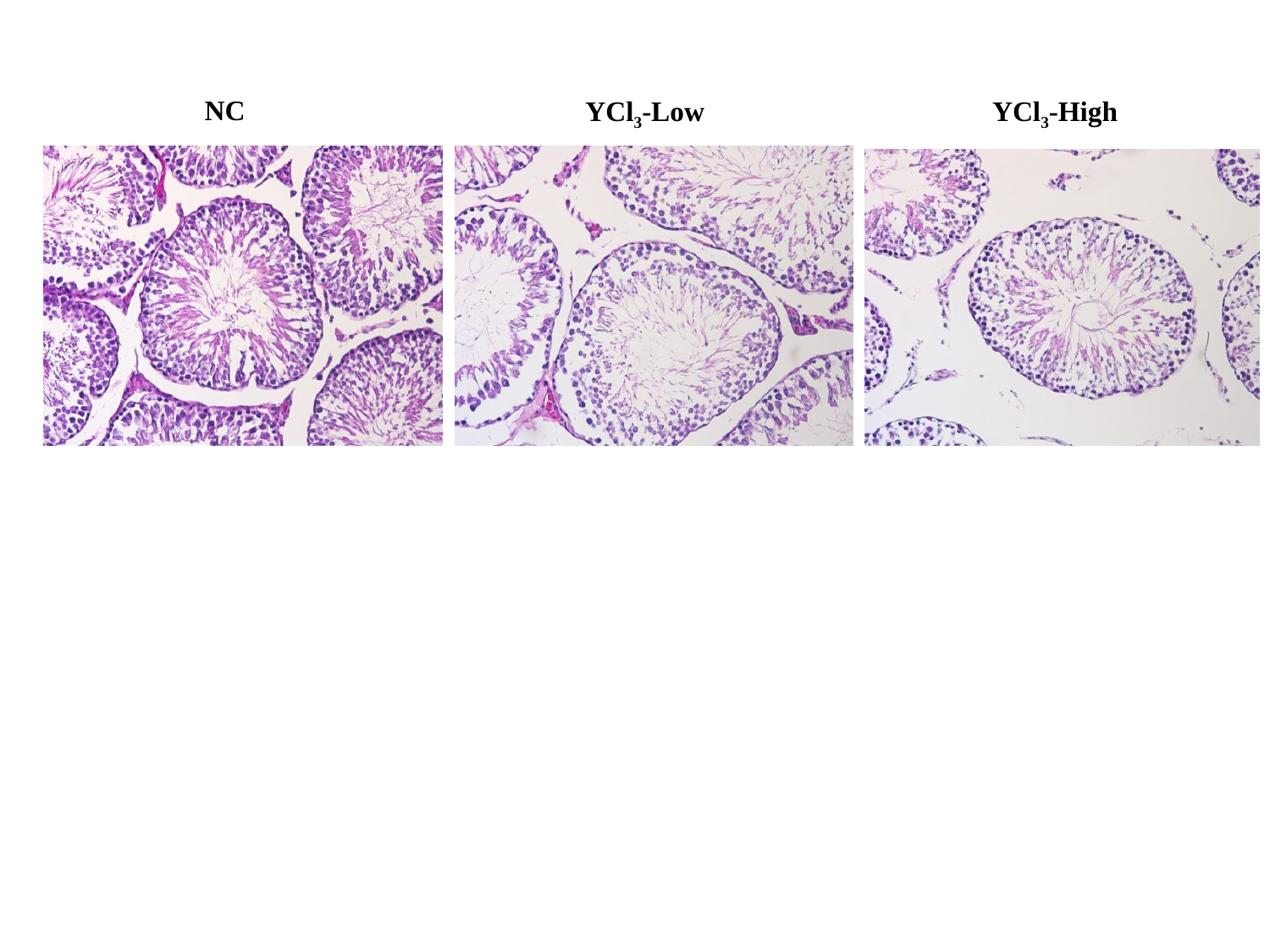

NC
YCl3-Low
YCl3-High

## Slide 2
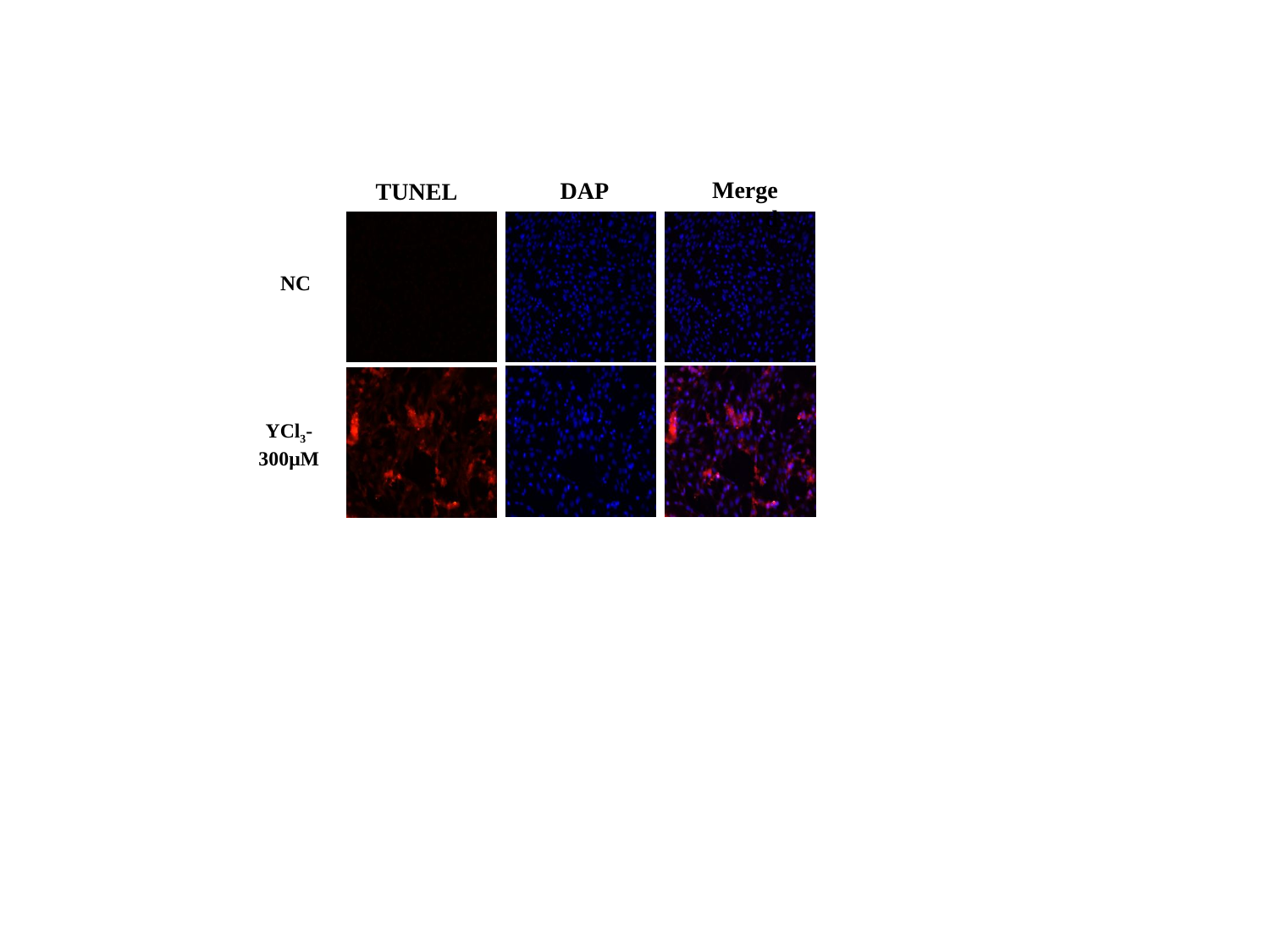

Merged
DAPI
TUNEL
NC
YCl3-
300μM

## Slide 3
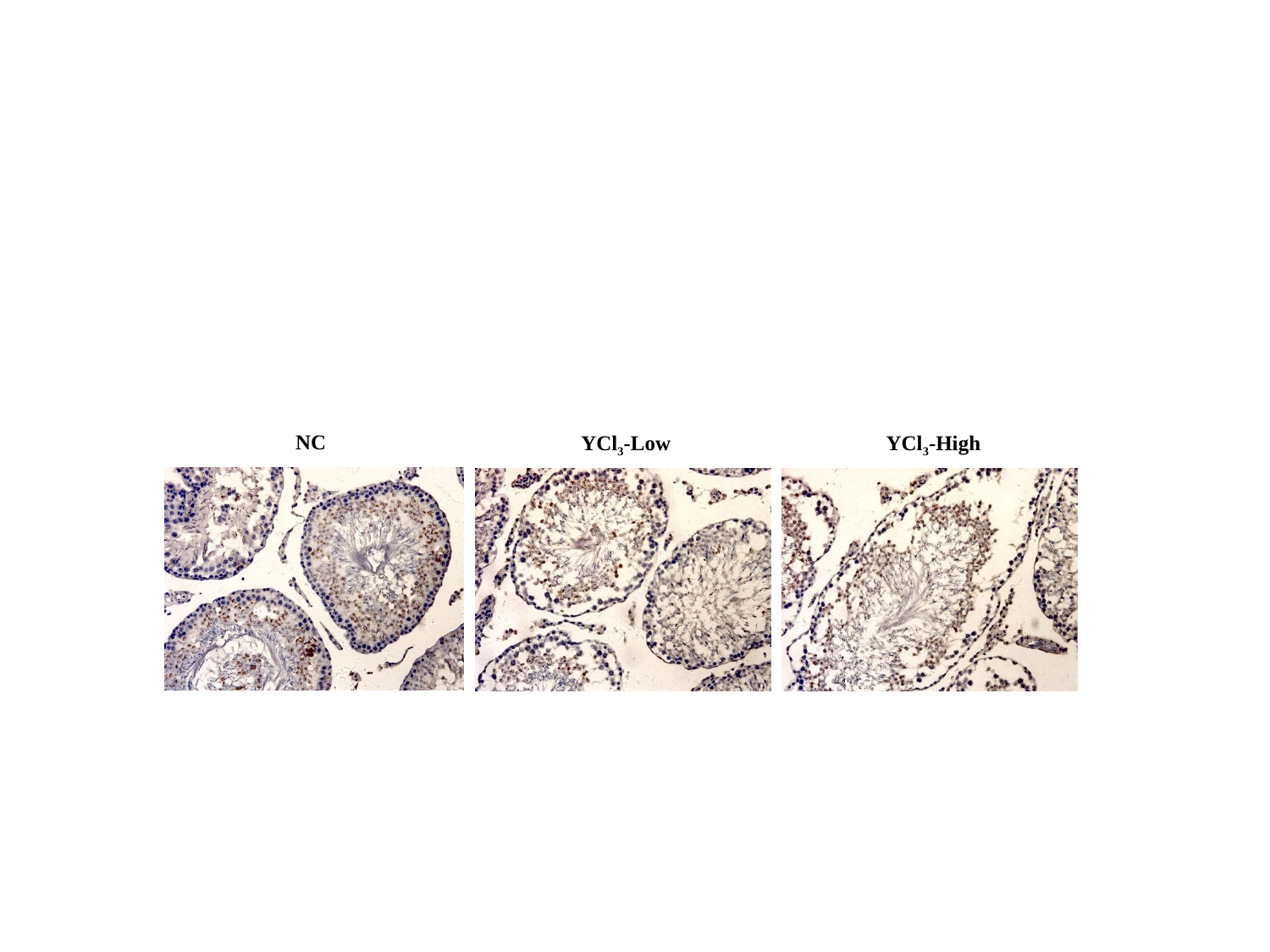

NC
YCl3-Low
YCl3-High

## Slide 4
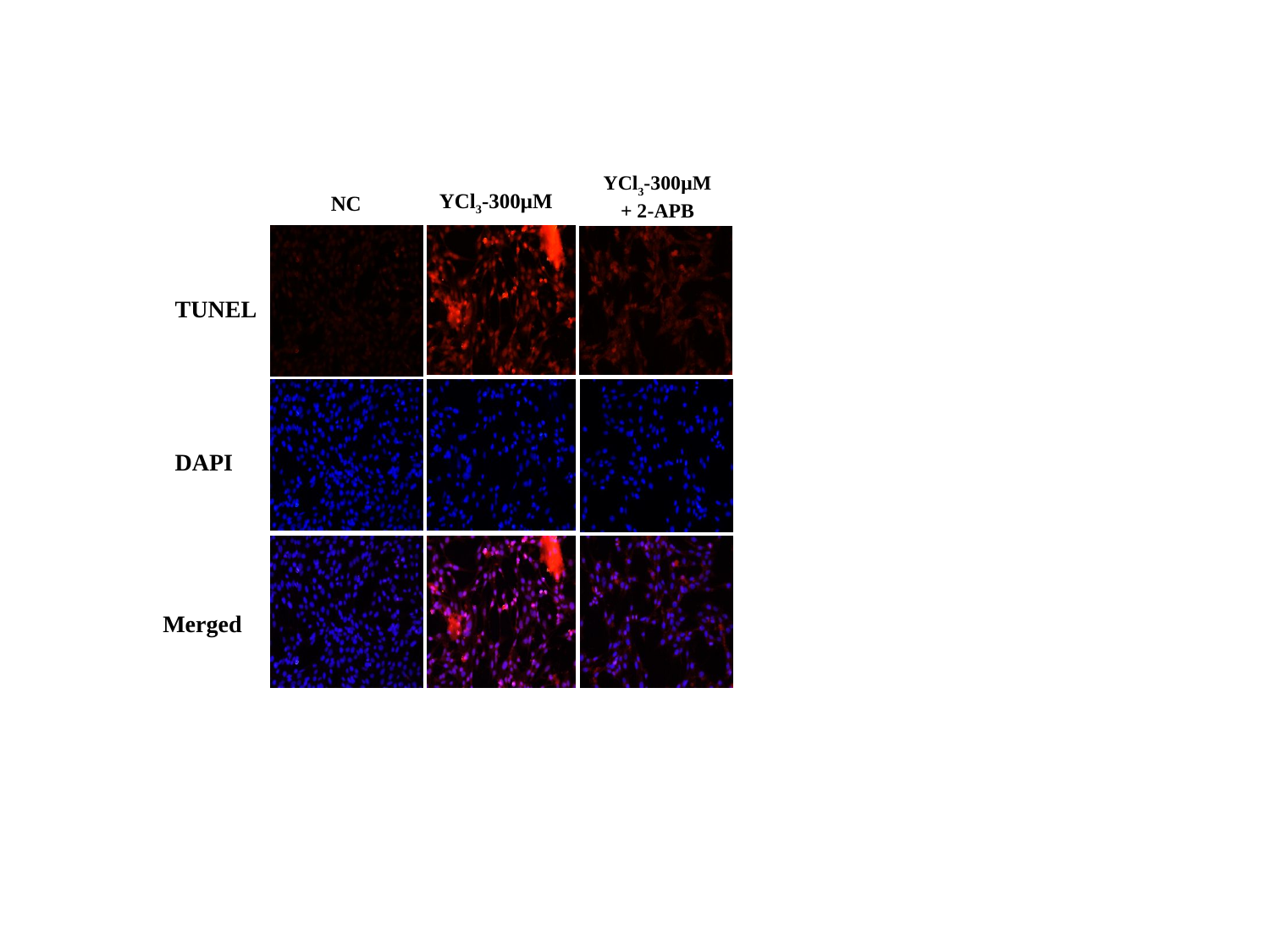

YCl3-300μM
+ 2-APB
YCl3-300μM
NC
TUNEL
DAPI
Merged

## Slide 5
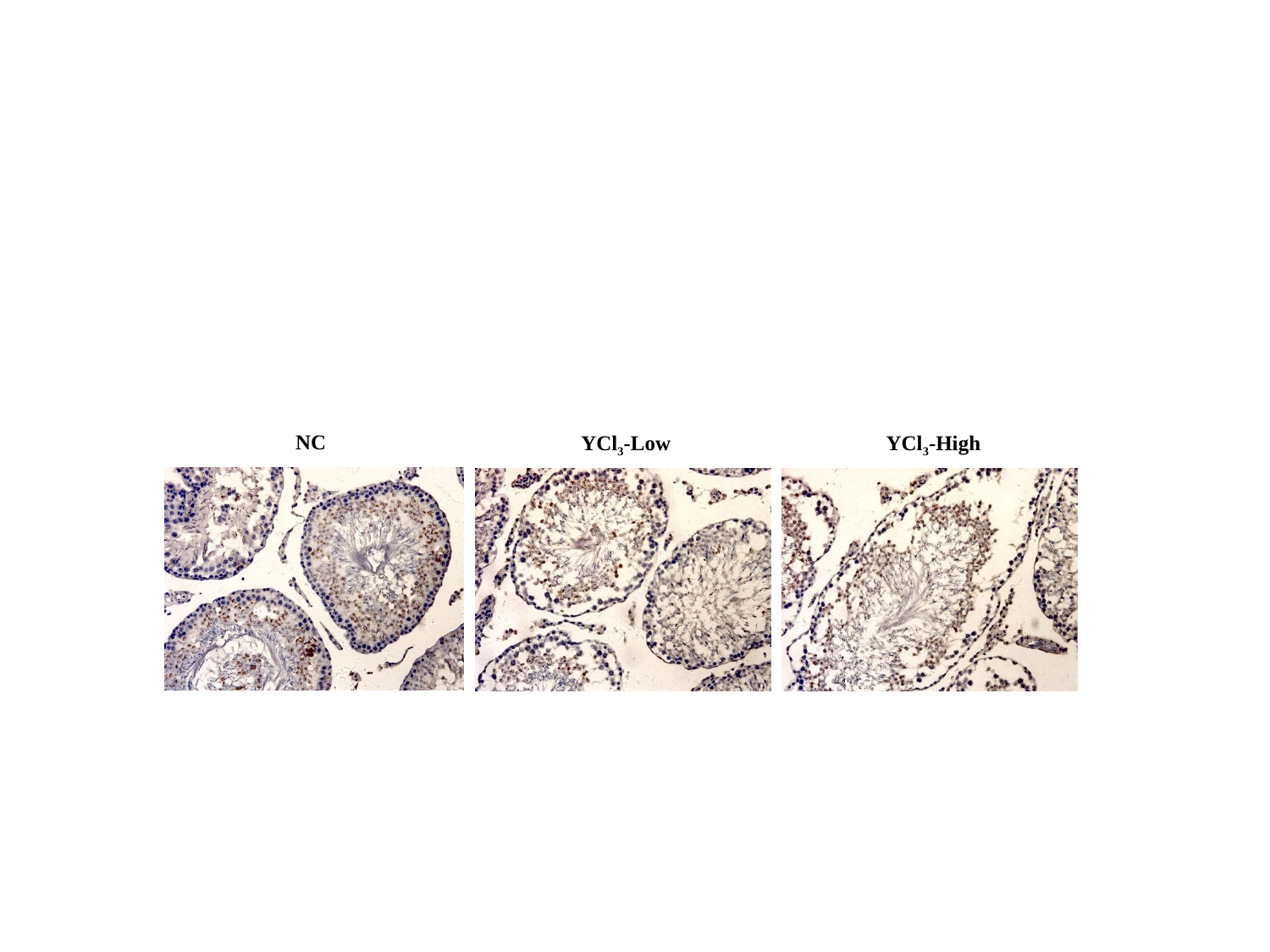

NC
YCl3-Low
YCl3-High

## Slide 6
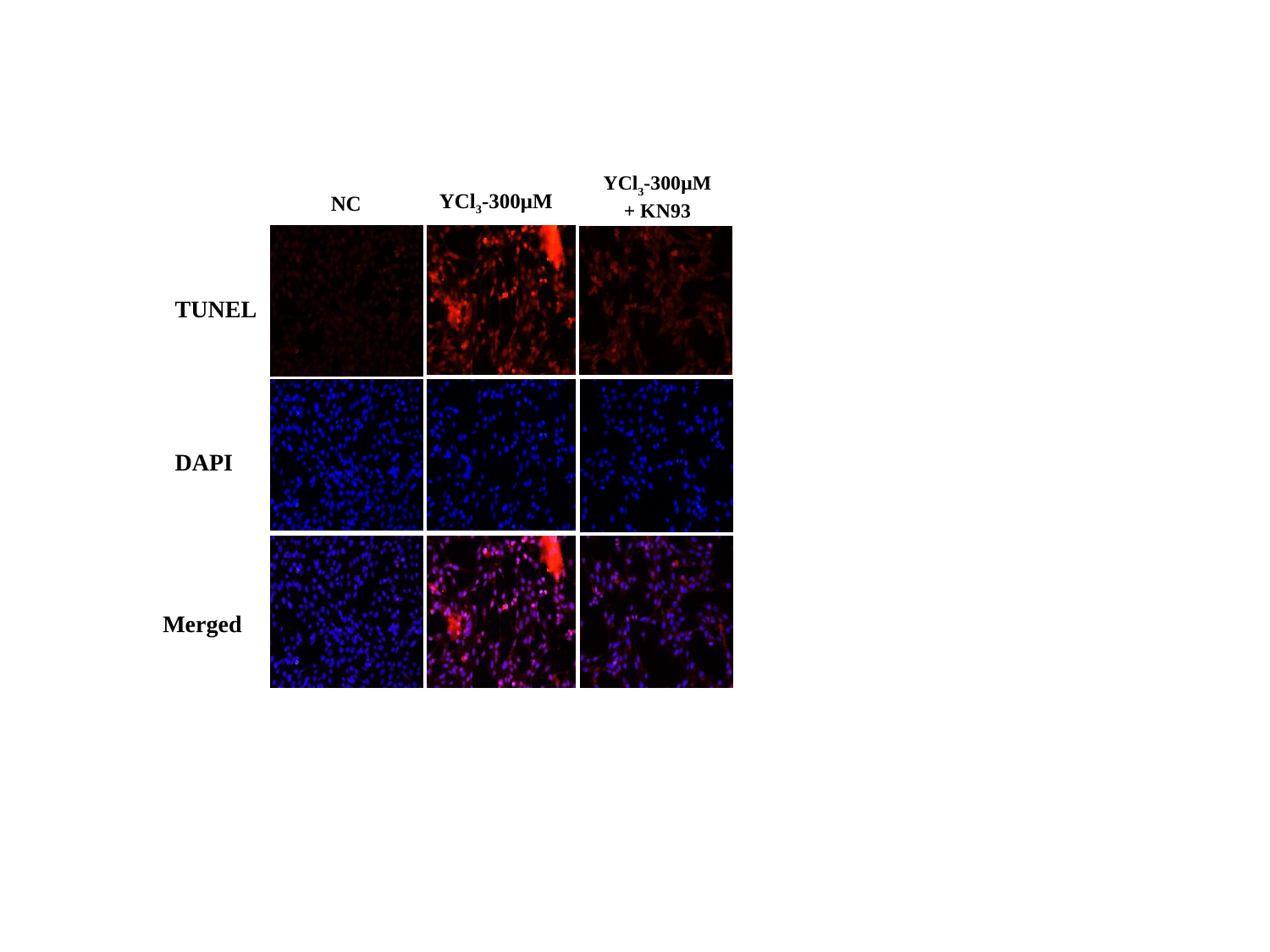

YCl3-300μM
+ KN93
YCl3-300μM
NC
TUNEL
DAPI
Merged
